# Treatment of Pulmonary Sequestrations by Means of Endovascular Embolization: Future or Fashion?

**DOI:** 10.1155/2011/173918

**Published:** 2011-09-22

**Authors:** Jeroen Diks, P. Ronald Schütte, David Cheung, J. Marco Schnater

**Affiliations:** Department of Surgery, Albert Schweitzer Hospital, P.O. Box 444, 3300 AK Dordrecht, The Netherlands

## Abstract

Bronchopulmonary sequestration is a rare malformation of the lower respiratory tract. Several methods of treatment have been described since the first publication. We present two cases of female adult patients with bronchopulmonary sequestration. In the first patient an unsuccessful attempt to treat the bronchopulmonary sequestration by means of arterial embolization is described. She was subsequently treated by means of surgical resection, which was the primary treatment for the second patient. Although endovascular techniques are becoming promising, in our opinion surgical resection remains the unique treatment for bronchopulmonary sequestration.

## 1. Introduction

Bronchopulmonary sequestration (BPS) is a rare congenital malformation of the lung tissue. It consists of normal lung tissue, which is separated from the normal tracheobronchial tree. Therefore, it does not contribute to the process of blood oxygenation. 

As a rare affection, it is estimated to consist of 0.15 to 6.4 percent of all congenital pulmonary malformations [1], affecting both males and females about equally. Although several options for treatment of BPS are described since its first reports in 1949 [2], the gold standard remains surgical resection [3]. 

We report two cases describing our experience with both arterial embolization and surgical resection. 

### 1.1. Case  1

**Figure 1:**
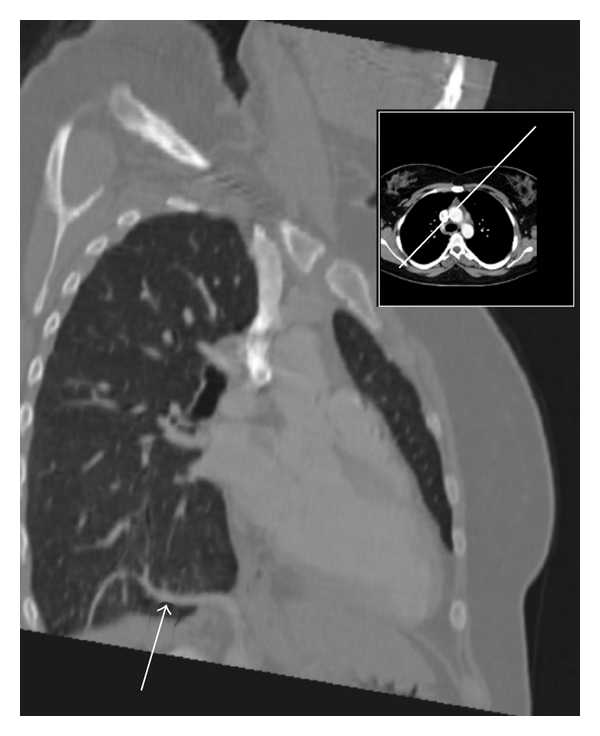
Aberrant arterial supply (white arrow) to intralobar pulmonary sequestration in right lower lung.

A 25-year-old patient reported to our outpatient clinic with a history of acute onset hemoptysis and chest pain on inspiration. Physical diagnostics as well as plain chest radiography were without irregularities. A high-resolution CT-angiography of the thorax and upper abdomen showed an aberrant artery originating from the abdominal aorta, which delivered vascular supply to an intralobar pulmonary sequestration in the right lower lung (Figure 1). 

After careful consideration in an interdisciplinary meeting, an embolization of the aberrant artery of the pulmonary sequestration was performed. The day after embolization, our patient presented to our emergency department with acute thoracic pain (Visual Analogue Scale 10) and respiratory distress. Thoracic X-ray showed no pneumothorax and CT scan showed no pulmonary embolism. She was admitted to our ICU with the diagnosis “pleural pain after embolization.” Analgesics were initially given by means of intravenous morphine-perfusion and, due to insufficient effect, subsequently by means of a thoracic epidural catheter. After initial recovery, she endured an episode of aggravation of symptoms, and we decided to perform a video-assisted thoracoscopic surgery (VATS) segmental resection. Histological findings showed pulmonary sequestration with signs of an old bleeding, no indication of malignancy or infection.

The patients' postoperative course was uneventful. However, four months after surgery, our patient presented to our pain clinic with persistent pain of the right hemithorax. She was diagnosed with intercostal neuralgia and was treated with a pulsed radiofrequency of the 9th intercostal nerve. At followup after two months, our patient remained pain-free.

### 1.2. Case  2

**Figure 2:**
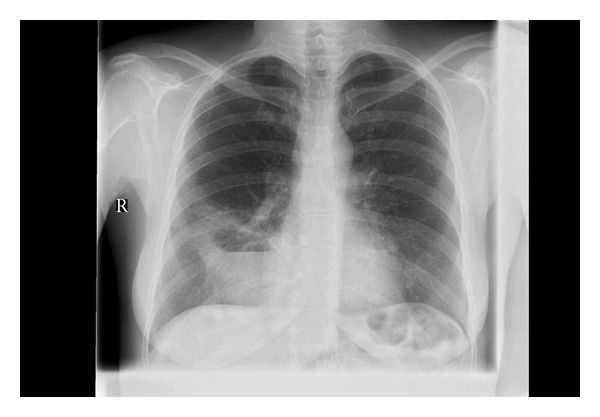
Abscess formation of an intralobar pulmonary sequestration in right lower lung.

A 50-year-old patient was seen at our outpatient clinic with recurrent pneumonias for which she was repeatedly treated with antibiotics. Chest radiography showed a consolidation in the right lower lobe with an air-fluid level (Figure 2). Subsequent CT angiography showed abscess formation of an intralobar pulmonary sequestration. The patient was treated by means of a thoracotomy with wedge-resection of two segments of the lower right lobe. 

Histological findings showed pulmonary sequestration with signs of infection and abscess formation and no signs of malignancy. 

Postoperatively our patient initially recovered well but developed a pneumonia after 10 days. This was treated successfully with intravenous antibiotics, and control CT angiography showed no signs of pleural empyema. Two weeks after surgery our patient could be discharged in good clinical condition. At followup after 6 months, she was well recovered and chest radiography showed no consolidations.

## 2. Discussion

A bronchopulmonary sequestration (BPS) consists of a section of nonfunctional pulmonary tissue, does not communicate with the normal bronchial tree, and is vascularized by an aberrant systemic artery. Its venous drainage is through the pulmonary veins, the azygos system, or the inferior vena cava [4]. Bronchopulmonary sequestrations are classified—according to their anatomy—into intralobar pulmonary sequestrations (IPS) or extralobar sequestrations (ELS). In an IPS, the sequester is located inside the normal pulmonary tissue. Its predominant site is the lower lobe, more often left than right. Although normally no communication with the bronchial tree exists, anomalous connection with other bronchi or lung parenchyma regularly occur. When this happens, recurrent infections are often seen [5]. 

Extralobar sequestrations are located inside their own visceral pleura and mostly found between the lower—foremost left—lobe and diaphragm or in the upper abdomen. There is utter separation with other lung tissue and therefore infections are uncommon. 

Patients with ELS usually show early signs of respiratory distress during childhood, while patients with ILS are more commonly diagnosed during adolescence or adulthood and show recurrent pneumonias or hemoptysis [6]. The diagnosis can be suspected on a chest radiograph, showing a dense mass or an air-fluid level. Best diagnostics, though, are obtained through a CT angiography, showing the aberrant artery and venous drainage [7, 8]. The gold standard for treatment of BPS is a surgical resection [3]. In ELS, a resection of the sequester is performed by means of video assisted thoracoscopic surgery (VATS) or a conventional thoracotomy. Both techniques can be used to perform a segmental resection or a lobectomy in ILS [9]. However, during the last decade a new technique has emerged. Arterial embolization as a treatment of arterial bleedings has rapidly become the treatment of the first choice for a wide range of indications. This approach to successfully treating BPS has been reported since 1998 [10]. In spite of these positive reports, we were unable to effectively treat our patient by use of this technique. Even though selective embolization of the aberrant artery was successfully performed—as confirmed by postoperative imaging—our patient endured an episode of extreme pleural pain for which we could find no other evident cause and she was ultimately treated by means of VATS. 

After this inadvertent result, questions raised as whether to primarily treat such patients with endovascular techniques. After a second patient presented with IPS, we proceeded with a thoracotomy rather than an embolization. Clinical outcome of the surgical approach was successful in both cases. This experience emphasizes our conviction that surgical resection remains the unique treatment for bronchopulmonary sequestration. It is the only treatment in which the abnormal lung tissue gets totally removed thus giving no opportunity for complications like ischemic infarction or abscess formation.
